# Brain-Immune Alterations and Mitochondrial Dysfunctions in a Mouse Model of Paediatric Autoimmune Disorder Associated with Streptococcus: Exacerbation by Chronic Psychosocial Stress

**DOI:** 10.3390/jcm8101514

**Published:** 2019-09-20

**Authors:** Maria Antonietta Ajmone-Cat, Chiara Spinello, Daniela Valenti, Francesca Franchi, Simone Macrì, Rosa Anna Vacca, Giovanni Laviola

**Affiliations:** 1 National Center for Drug Research and Evaluation, Istituto Superiore di Sanità, Viale Regina Elena, 299, I-00161 Rome, Italy; mariaantonietta.ajmone-cat@iss.it; 2 Centre for Behavioural Sciences and Mental Health, Istituto Superiore di Sanità, Viale Regina Elena, 299, I-00161 Rome, Italy; chiara.spnll@gmail.com (C.S.); francesca.franchi@iss.it (F.F.); simone.macri@iss.it (S.M.); 3 Department of Mechanical and Aerospace Engineering, New York University Tandon School of Engineering, Brooklyn, NY 11201, USA; 4 Institute of Biomembranes, Bioenergetics and Molecular Biotechnologies, National Council of Research, Via Giovanni Amendola 122/O - 70126 Bari, Italy; d.valenti@ibiom.cnr.it

**Keywords:** PANDAS, adverse emotional experience, immunity, neuroinflammation, animal Models, mitochondrial bioenergetics

## Abstract

Adverse psychosocial experiences have been shown to modulate individual responses to immune challenges and affect mitochondrial functions. The aim of this study was to investigate inflammation and immune responses as well as mitochondrial bioenergetics in an experimental model of Paediatric Autoimmune Neuropsychiatric Disorders Associated with Streptococcus (PANDAS). Starting in adolescence (postnatal day 28), male SJL/J mice were exposed to five injections (interspaced by two weeks) with Group-A beta-haemolytic streptococcus (GAS) homogenate. Mice were exposed to chronic psychosocial stress, in the form of protracted visual exposure to an aggressive conspecific, for four weeks. Our results indicate that psychosocial stress exacerbated individual response to GAS administrations whereby mice exposed to both treatments exhibited altered cytokine and immune-related enzyme expression in the hippocampus and hypothalamus. Additionally, they showed impaired mitochondrial respiratory chain complexes IV and V, and reduced adenosine triphosphate (ATP) production by mitochondria and ATP content. These brain abnormalities, observed in GAS-Stress mice, were associated with blunted titers of plasma corticosterone. Present data support the hypothesis that challenging environmental conditions, in terms of chronic psychosocial stress, may exacerbate the long-term consequences of exposure to GAS processes through the promotion of central immunomodulatory and oxidative stress.

## 1. Introduction

Recent epidemiological, clinical, and preclinical studies suggest that immune responses to pathogens may play a remarkable role in the onset and course of a heterogeneous group of psychiatric disturbances. Several authors proposed that genetically predisposed individuals may develop a series of immune-mediated disturbances, ranging from Paediatric Autoimmune Disorders Associated with Streptococcus (PANDAS) to neuropsychiatric systemic lupus erythematosus and autoimmune encephalopathies [1,2,3,4]. Within this framework, pathogen-directed antibodies are hypothesized to cross-react with a variety of partly identified neuronal antigens for a phenomenon of molecular mimicry, thereby damaging specific brain circuits and ultimately result in behavioural abnormalities [5,6].

The acronym PANDAS was coined to define a series of disturbances with a paediatric onset—mostly characterized by the exhibition of choreic or repetitive movements—in which repeated exposures to bacterial infections (in particular of Group-A beta-Haemolytic Streptococcus, GAS) are causally linked to the exhibition of symptoms [7,8,9,10,11,12]. Recurrent exhibition of abnormal behaviours, and remitting-relapsing presence of obsessive-compulsive symptoms and/or tics are among the defining criteria of PANDAS [13,14]. Sydenham’s chorea (SC) and Tourette’s syndrome (TS) have been proposed to constitute instances of PANDAS [15,16].

Orlovska and co-authors provided additional support to the PANDAS hypothesis through a cohort study wherein they observed that individuals with streptococcal throat infection had elevated risks of obsessive-compulsive (OCD) and tic disorders [17]; non-streptococcal throat infection was also associated with increased risks, albeit to a lesser extent, suggesting an even wider association between immunity and neurological sequelae.

The possibility that autoimmune phenomena may contribute to the manifestation of neurological and behavioural disturbances has been corroborated by several independent preclinical studies [9,12,18]. Hoffman and colleagues demonstrated that mice repeatedly injected with Streptococcus homogenate showed locomotor alterations associated with IgG deposits in deep cerebellar nuclei [9]. We recently extended these observations and reported that analogous treatments in mice resulted, in the long term, in increased repetitive behaviours, impairments in sensorimotor gating, and indices of inflammatory processes occurring at the level of the rostral diencephalon [18,19]. 

Symptom fluctuations and recurrences in PANDAS seem to be influenced by various contextual features (see [13,20] for reviews), among which psychosocial factors and stress have called particular attention [21,22,23]. Environmental stressors contribute to vulnerability to Tourette’s syndrome, and play a remarkable role in modulating the severity of clinical symptoms [24,25]. Similarly, acute psychosocial stressors have been associated with worsening of tics [20,26,27]. On the other hand, chronic psychosocial stress has been shown to influence individual adaptation to additional challenges [28].

In a translational rodent model of repeated GAS injections [19], we originally reported that neonatal exogenous corticosterone administration mitigated behavioural and immunohistochemical alterations induced by subsequent GAS injections. These compensatory effects co-occurred with modifications in hypothalamic pituitary adrenal axis (HPA) activity and remarkable increases in plasma inflammatory cytokines and chemokines. These results support the view that the HPA axis may contribute to the regulation of the immune responses involved in the pathological sequelae of PANDAS and ultimately modulate the severity of the PANDAS-related phenotype [19].

Stress and highly demanding social dynamics have been causally linked to both mental and somatic pathologies [29,30,31,32,33,34,35], albeit the underlying mechanisms remained elusive.

Compelling evidence indicate stress-mediated alterations in HPA activity may influence the immune system, favour peripheral inflammation as well as neuroinflammation [36], increase individual vulnerability towards subsequent immune challenges, and ultimately promote autoimmunity [37,38,39,40]. Different psychogenic stressors induce oxidative and nitrosative stress in the central nervous system (CNS) and few studies showed that the development of psychosis in immune activation translational models could be mediated by an imbalance between pro-oxidants and anti-oxidants [41]. Accordingly, several clinical, genetic, and biochemical studies highlighted a central role of impaired mitochondrial function and oxidative stress in the etiology of both neurological and neuropsychiatric diseases [42,43,44]. Just as the immune system represents a major source of oxidative stress, so also oxidative stress induces inflammation via the activation of nuclear factor κB (NF-κB), a key transcription factor for the modulation of inflammatory genes [41]. It is well known that dysfunction of the mitochondrial respiratory chain (MRC) machinery leads to a decrease in adenosine triphosphate (ATP) production through oxidative phosphorylation (OXPHOS) (for refs see [45]) and exposure to oxidative stress could exacerbate mitochondrial damage and induce death at the cellular level [46]. Beside regulating cellular energy-generating processes, mitochondria play a pivotal role in controlling immune cell activation and functions (see [47,48,49,50]), and may thus represent targets of inflammatory cytokines in immune-mediated diseases [51]. 

In the present study, we aimed at investigating the role of social stressors in the calibration of psychiatric disorders, and at identifying candidate mediators potentially serving as future therapeutic targets. Specifically, we aimed at: (i) clarifying the role of the immune system in the pathological sequelae linking streptococcal infections and psychiatric disturbances; (ii) providing additional evidence that psychosocial stress may exacerbate the symptoms occurring in response to streptococcal infection; (iii) demonstrating that mitochondrial oxidative phosphorylation apparatus may represent a candidate biological determinant, potentially representing a valid therapeutic target. 

Based on the evidence discussed above, we evaluated the possibility that adverse environmental factors calibrate individual vulnerability in a validated translational mouse model of PANDAS [18,19]. To this aim, we tested the prediction that chronic psychosocial stress may worsen the brain immune response and induce oxidative stress and mitochondrial dysfunction. In the present study, we opted to focus on male mice due to the higher incidence of PANDAS observed in males compared to females (with a 4.7:1 ratio; see [8]). While we acknowledge that it would be important to extend our findings to females, we note that in the light of the innovative nature of this study, it was necessary to identify a proof-of-principle in the most promising study population (males) before planning a study involving female subjects.

## 2. Experimental Section

### 2.1. Animals and Rearing

Male SJL/J mice, postnatal day (PND) 25 on the day of arrival, were purchased from Charles River, Italy (Calco, Lecco, Italy). Upon arrival, mice were randomly housed in groups of two individuals in type-1 polycarbonate cages (33 × 13 × 14 cm). All cages were equipped with sawdust bedding, an enrichment bag (Mucedola, Settimo Milanese, Italy), metal top and ad libitum water and food pellets (Mucedola, Settimo Milanese, Italy). Mice were maintained on a reversed 12-h-light-dark cycle (light on at 7:00 PM) in an air-conditioned room (temperature 21 ± 1 °C and relative humidity 60 ± 10%). 

All experimental procedures were performed in agreement with the Legislative Decree 26/14 and the European Directive 2010/63/UE on laboratory animal protection and experimentation. The study has been approved by the Italian Ministry of Health (Decree Nr. 217/2010-B).

### 2.2. Immunization Protocol

GAS homogenate was prepared as described in previous studies [9,18,19]. After preparation, streptococcus homogenate was stored at −70 °C. A blood agar plate was used to inoculate a sample of homogenate (2.5 µL), to verify that it contained no viable bacteria. Immunization protocol, described in [9] and adopted in our previous study [18], comprised five injections interspaced by a time interval of two weeks, starting on PND 28. During the first injection, Phosphate-buffered saline (PBS) mice were injected subcutaneously (s.c.) with 125 µL of an emulsion (1:1), containing PBS and Complete Freund’s adjuvant (CFA; Sigma Aldrich, Milano, Italy), and GAS mice were injected with 125 µL of the same emulsion (PBS:CFA), containing 5 µL of GAS homogenate (0.52 mg/mL of total protein as determined by Bradford Assay, Biorad, (Hercules, CA, USA). Mice were then treated four additional times at 2-week intervals with 125 µL of vehicle – an emulsion (1:1) containing PBS and Incomplete Freud’s Adjuvant (IFA; Sigma Aldrich, Milano, Italy) – for the PBS group, or 125 µL of PBS:IFA and 5 µL of GAS homogenate for the GAS group. To prepare the PBS/adjuvant emulsions we used the vortex method described by [52]. According to the adult and the PBS/GAS treatment received, experimental subjects were randomly assigned to four experimental groups: control vehicle-injected (PBS-no Stress), GAS homogenate/vehicle-injected (GAS-no Stress), vehicle-injected plus psychosocial stress (PBS-Stress), and GAS homogenate/vehicle-injected plus psychosocial stress (GAS-Stress). 

### 2.3. Chronic Psychosocial Stress

Psychosocial stress consisted of a 4-week protocol in which mice were exposed to daily defeats and sensory contact housing (enabled by a wire mesh partition bisecting the cage into two symmetrical compartments, each with food and water available at libitum). The chronic psychosocial stress, starting at PND 56, was conducted as previously described [53,54]. Briefly, each SJL male mouse, representing the experimental subject, was transferred as intruder to the home cage of a CD1 resident mouse. The CD1 strain manifests high territorial aggression [55]. Resident and intruder mice were allowed to freely interact for a maximum of 10 min during the 1st social confrontation. After the interaction, resident and intruder mice were separated by a perforated partition, which allowed continuous sensory contact but no physical interaction. The partition was removed daily (between 9:30 AM and 12:30 PM or between 2:30 PM and 5:00 PM), for a theoretical maximum of 10 min. However, from the 2nd event onwards, because of the elevated level of aggressive behavior displayed by the resident mouse, the physical confrontation was generally interrupted shortly after its beginning. During the first active social interaction, offensive/defensive behaviors and the display of upright posture, flight behavior of the experimental SJL mice were video recorded for subsequent scoring [55,56]. Videos were scored by a trained observer using a specific software (The Observer, The observer XT 10, Noldus, PA Wageningen, The Netherland). Behaviors observed are part of the aggressive and defensive/subordination repertoire of male mice [57,58]. In particular, the behaviors collected were: attack (forward motion of the resident mouse toward the mouse belonging to the experimental group; the motion is combined with direct physical contact), defensive upright (the animal stands on the hind-limbs and push the aggressive opponent with the forepaws, the head pulled far back), fleeing (the animal rapidly escapes from the opponent, often screaming), immobility-attack related (the animal is motionless during an attack), immobility-contact related (the animal is motionless while the opponent is in physical contact but is not attacking) and submissive behaviors (animal standing on its hind limbs while having the head pulled far back; also, the body is rigid). Furthermore, self-grooming (the mouse licks its own fur helping itself with its forepaws) were scored. 

### 2.4. Experimental Design

**Figure 1:**
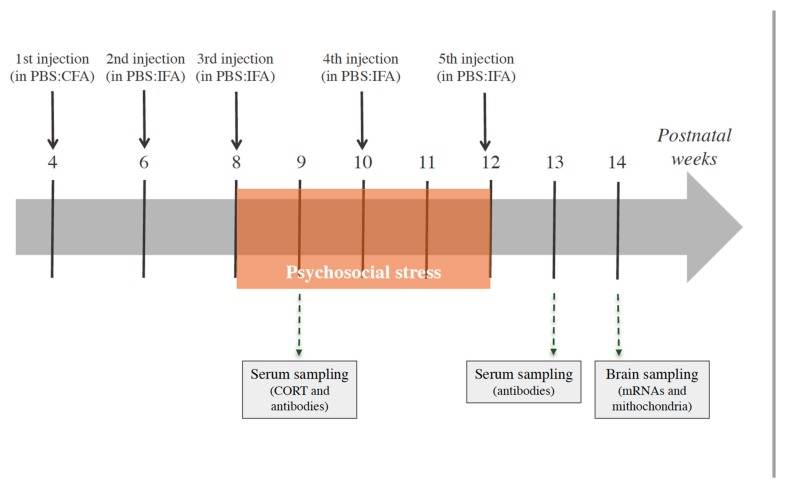
Timing of stress exposure and of the Group-A beta-haemolytic streptococcus (GAS) injections, expressed in weeks, and the experimental procedures performed. Mice (N = 9–10 per group) received 5 injections of GAS homogenate or Phosphate Buffer Saline (PBS), formulated with the indicated adjuvants (CFA = Complete Freund’s adjuvant; IFA = Incomplete Freund’s adjuvant). Serum samples were collected for antibody determination and corticosterone (CORT) concentration assessment; brain samples were collected for mRNA expression and mitochondrial analyses.

In the four experimental groups of mice consisting of PBS-no Stress (N = 10), GAS-no Stress (N = 9), PBS-Stress (N = 10) and GAS-Stress (N = 10), the experimental design (Figure 1) comprised the following evaluations: the immune response to the injection protocol and corticosterone determination in blood sampling; assessment of neuroinflammatory and mitochondrial parameters in brain sampling/sectioning.

### 2.5. Blood Serum Sampling and Corticosterone Determination 

To evaluate the effects of experimental treatments on HPA activity, we evaluated serum corticosterone concentrations at post-natal week 9, one week after the beginning of the psychosocial stress procedure. Blood samples (~20 µL) were collected through tail incision [59,60] at 9:00 PM, i.e. two hours after the beginning of the dark phase of the inverted light-dark cycle, and before the beginning of the daily stress procedure. Blood samples were allowed to clot at room temperature for 4 hours, centrifuged at 3000 rpm for 15 minutes. The serum was transferred into Eppendorf tubes and maintained at −80 °C until biochemical assays. Corticosterone concentration was assessed using a commercial radioimmunoassay (RIA) kit (ICN Biomedicals, Costa Mesa, CA, USA). Vials were counted for 2 minutes in a gamma counter (Packard Minaxi Gamma counter, Series 5000, Packard Instruments Company Inc, Meriden, CT, USA). The procedures for washing and steroid extraction followed the protocol described by Gao and colleagues [61]. One change was made to the protocol: the dry residue was resuspended using 175 µL distilled water. Afterwards, 100 µL of the medium were injected into a Shimadzu HPLC system (Shimadzu, Canby, OR, USA) coupled to an AB Sciex API 5000 Turboion-spray1triple quadrupole tandem mass spectrometer equipped with Atmospheric Pressure Chemical Ionization (APCI) Source (AB Sciex, Foster City, CA, USA). The system was controlled by AB Sciex Analyst1 software (version 1.5.1, AB Sciex, Milano, Italy). The lower limit of detection was ~0.1 pg/mg. Intra- and inter-plate coefficients of variance ranged between 3.7–8.8%. All samples were prepared and analysed within the same time period in order to prevent batch effects.

### 2.6. Analysis of Anti-Group A Streptococcal Antibodies in Serum Samples 

GAS homogenates obtained as described above (see Section 2.2) were size-separated by SDS-PAGE (4–12% acrylamide) under reducing conditions and electroblotted onto nitrocellulose membranes. Immunostaining was performed by blocking the membrane overnight with 3% (*w*/*v*) skimmed milk in TPBS (0.1% Tween in PBS) and incubating for 2 h with sera from mice of all experimental groups (all sera were diluted 1: 200). For this analysis, sera were collected one week after the 3rd and 5th injection of GAS homogenate or adjuvant alone following the procedure described above (see Section 2.5). After 3 washes with TPBS, the membrane was incubated with Horseradish Peroxidase (HRP)-conjugated secondary antibody (1:1000), washed again with TPBS and PBS, and developed with a chromogenic substrate. Densitometric analysis of the overall lane intensities in the blot was performed by using Image J software (Image J2, Image J developers). 

### 2.7. Brain Sampling

In order to collect brain samples, mice were rapidly decapitated, two weeks after treatment endings. This time point was chosen to investigate the possible long-term effects of treatments on brain immune and mitochondrial parameters. Samples collected were immediately sectioned on ice to obtain hippocampus, and hypothalamus. For mRNA expression analyses, brain samples were collected after decapitation, kept intact in mRNAase free tubes, flash frozen and stored at −80 °C. 

For mitochondrial analysis, brain hemispheres immediately after explant were added to an ice-cold cryopreservation solution consisting of 50 mM K-MES (pH 7.1), 3 mM K_2_HPO_4_, 9.5 mM MgCl_2_, 3 mM ATP plus 20% glycerol and 10 mg/mL BSA, and stored at −80 °C until assayed. We have previously demonstrated that mitochondria isolated from cryopreserved brain tissues show mitochondrial membrane potential, outer and inner membrane integrity and mitochondrial ATP production capacity comparable to mitochondria isolated from fresh brains [62,63].

### 2.8. Real-Time Quantitative Polymerase Chain Reaction (RT-PCR)

Dissected hypothalami and hippocampi (from N = 6 mice per experimental group) were homogenized in Tri Reagent (Sigma, St. Louis, MO, USA) and mRNA extraction was performed on supernatants. 

Total RNA (1 μg) from each sample was transcribed into complementary DNA using the RT-PCR Superscript III kit (Invitrogen, Eugene, OR, USA), according to the manufacturer’s instructions. RT-PCR was performed on the reverse transcription products with a SensiMix SYBR Kit (Bioline, London, UK) for hypoxanthine guanine phosphoribosyl transferase (HPRT), tumor necrosis factor-α (TNF-α), interleukin-1β (IL-1β), interleukin-10 (IL-10), inducible nitric oxide synthase (iNOS), arginase-1 (Arg-1), manganese superoxide dismutase (MnSOD), and glucocorticoid receptor (GR) mRNA expression, or with TaqMan for HPRT and CD11b, using an ABI Prism 7500 Sequence Detection System (Applied Biosystems, Foster City, CA, USA). 

Primer sequences for HPRT, IL-1β, TNF-α, IL-10, iNOS, Arg-1, MnSOD, and GR were from Integrated DNA Technologies (IDT, TEMA Ricerca Bologna, Italy); accession numbers are as follows:

1. HPRT (NM_013556): forward 5′-CAGGCCAGACTTTG-TTGGAT-3′; reverse 5′-TTGCGCTCATC-TTAGGCTTT-3′;
2. IL-1β (NM_008361): forward 5′-CGACAAAATACCTGTGGCCT-3′, reverse 5′-TTCTTTGGGTATTCCTTGGG-3′;
3. TNF-α (NM_013693.3): forward 5′-AGCCCCCAGTCTGTATCCTT-3′, reverse 5′-ACAGTCCAGGTCACTGTCCC-3′;
4. IL-10 (NM_010548): forward 5′-TTAAGCTGTTTCCATTGGGG-3′, reverse 5′-AAGTGTGGCCAGCCTTAGAA-3′;
5. iNOS (NM_010927): forward 5′-CAGCTGGGCTGTACAAACCTT-3′, reverse 5′-CATTGGAAGTGAAGCGTTTCG-3′;
6. Arg-1 (NM_007482): forward 5′-GGAAAGCCAATGAAGAGCTG-3′, reverse 5′-AACACTCCCCTGACAACCAG-3′;
7. MnSOD (NC_000083.6): forward 5′-GCTCTGGCCAAGGGAGATGT-3′, reverse, 5′-GGGCTCAGGTTTGTCCAGAAA-3′;
8. GR (NM_008173.3): forward 5′-CGCCAAGTGATTGCCGC-3′, reverse 5′-TGTAGAAGGGTCATTTGGTCATCCA-3′.

TaqMan primers for HPRT (Mn.PT.39a22214828) and CD11b (Mn.PT.58.9189361), were also from IDT.

Annealing temperature was 60 °C for all the primer pairs listed. All samples were run in triplicate, and each PCR well contained 20 μL as a final volume of reaction, including 2 μL complementary DNA corresponding to approximately 60 ng total RNA, 750 nM of each primer, and 10 μL PCR master mix. Thermal cycling conditions were as follows: 1 cycle at 95 °C for 10 min, 40 cycles at 95 °C for 15 s, and 60 °C for 1 min. The relative expression level of each mRNA was calculated using the ΔΔCt method normalized to HPRT and relative to the control samples. The amplification specificity was verified by melting curve analyses.

### 2.9. Measurement of Mitochondrial Respiratory Chain Complex (MRC) Activities

Measurements of mitochondrial respiratory chain (MRC) complex activities were carried out in mitochondrial membrane-enriched fractions obtained from crude mitochondria isolated by differential centrifugation of brain homogenate as previously described [62]. To obtain mitochondrial membrane-enriched fractions, mitochondrial pellets were first frozen at −80 °C, then thawed at 2–4 °C, suspended in 1 ml of 10 mM Tris-HCl (pH 7.5) plus 1mg/ml BSA and exposed to ultrasound energy for 8 s at 0 °C (11 pulse 0.7 s on, 0.7 s off) at 20 kHz, intensity 2. The ultrasound-treated mitochondria were centrifuged at 600 g for 10 min, 4 °C. The supernatant was centrifuged again at 14000 g for 10 min, 4 °C and the resulting pellet was kept at −80 °C until use. The MRC complex activities were assessed spectrophotometrically essentially as in [64], by three measurements which rely on the sequential addition of reagents to measure the activities of: (i) NADH:ubiquinone oxidoreductase (complex I) followed by ATP synthase (complex V), (ii) succinate:ubiquinone oxidoreductase (complex II) and (iii) cytochrome c oxidase (complex IV) followed by cytochrome c oxidoreductase (complex III).

### 2.10. Measurement of Mitochondrial ATP Production Rate

The rate of ATP production by OXPHOS was determined in isolated mitochondria, essentially as previously described in [65]. Briefly, mitochondria isolated from total brain (0.5 mg protein) were incubated at 37°C in 2 mL of respiratory medium consisting of 210 mM mannitol, 70 mM sucrose, 20 mM Tris/HCl, 5 mM KH_2_PO_4_/K_2_HPO_4_, (pH 7.4) plus 5 mg/mL BSA, 3 mM MgCl_2_, in the presence of the ATP detecting system consisting of glucose (2.5 mM), hexokinase (HK, 2 enzymatic units, e.u.), glucose 6-phosphate dehydrogenase (G6P-DH, 1 e.u.) and NADP^+^ (0.25 mM) in the presence of glutamate (GLU) plus malate (MAL) (5 mM each) or succinate (SUCC, 5 mM) plus rotenone (ROT, 3 µM), or ascorbate (ASC, 0.5 mM) plus *N,N,N′,N′*-tetramethyl-p-phenylenediamine (TMPD, 0.25 mM), as energy sources. The reduction of NADP^+^ in the extramitochondrial phase, which reveals ATP formation from externally added adenosine diphosphate (ADP, 0.5 mM), was monitored as an increase in absorbance at 340 nm. Care was taken to use enough HK/G6P-DH coupled enzymes to ensure a non-limiting ADP-regenerating system for the measurement of ATP production. 

### 2.11. Measurement of Mouse Brain ATP Levels

The brain hemisphere was subjected to perchloric acid extraction as described in [66]. In brief, tissues were homogenized in 600 µL of pre-cooled 10% perchloric acid and then centrifuged at 14000 g for 10 min, 4 °C. The amount of tissue ATP was determined in KOH neutralized extracts by spectrofluorimetric measurements (with excitation wavelength of 334 and emission wavelength of 456 nm) following the formation of NADPH, which reveals ATP, in the presence of the ATP detecting system consisting of glucose (2.5 mM), hexokinase (HK, 2 e.u.), glucose 6-phosphate dehydrogenase (G6P-DH, 1 e.u.), and NADP^+^ (0.25 mM) [67].

### 2.12. T-Maze

Animals were screened for perseverative behaviours in the T-maze test through the same procedure adopted in our previous study [19]. The T-maze provides an inverse index of perseverative behaviour whereby, in this test, rodents have the natural tendency to alternate their choices in a binary-test paradigm (spontaneous alternation) [68]. The apparatus was an enclosed T-shaped maze, composed of three equally sized arms (50 × 16 cm). Mice performed ten sessions, in the housing room, during five consecutive days (2 sessions per day). The experimental session consisted of two choice trials, beginning with the mouse in the start compartment, facing the wall of the apparatus. Mice were allowed to explore the apparatus for a maximum of two minutes, or until it completed the trial (entering one of the two alternative arms). Immediately after the mouse entered one arm, such instance was scored as the first choice and the door of the arm was closed. After a few seconds, the animal was gently removed from the arm, placed again in the starting compartment, and allowed to perform a second-choice trial. If the subject entered the arm opposite to the previously chosen one, an instance of alternation was scored. The percentage of alternations (the number of alternations divided by the number of completed sessions times 100) was scored for each mouse. 

### 2.13. Statistical Analyses

All statistical analyses were conducted using the software Statview 5.0 (Abacus Concepts, Piscataway, NJ, USA). The experimental design entailed two between subject factors (psychosocial stress, two levels; and Treatment, two levels: PBS vs. GAS) and one within-subject factor (repeated measures with a variable number of levels, depending on the specific parameter). Thus, the general experimental model consisted of a 2 (psychosocial Stress) × 2 (PBS/GAS treatment) × k (repeated measurements) repeated measures ANOVA for split-plot designs. Tukey’s post-hoc tests were used for between-group comparisons and Cohen’s d factor to measure the effect size between groups. Data are expressed as mean ± SEM or SD were specified. Statistical significance was set at *p* < 0.05. 

### 2.14. Data Statement

All data set produced in the present study are available upon request.

## 3. Results

### 3.1. Evaluation of Anti-GAS Antibody Responses in Sera from Mice Injected with GAS Homogenates and Exposed (or not) to Chronic Psychosocial Stress

**Figure 2:**
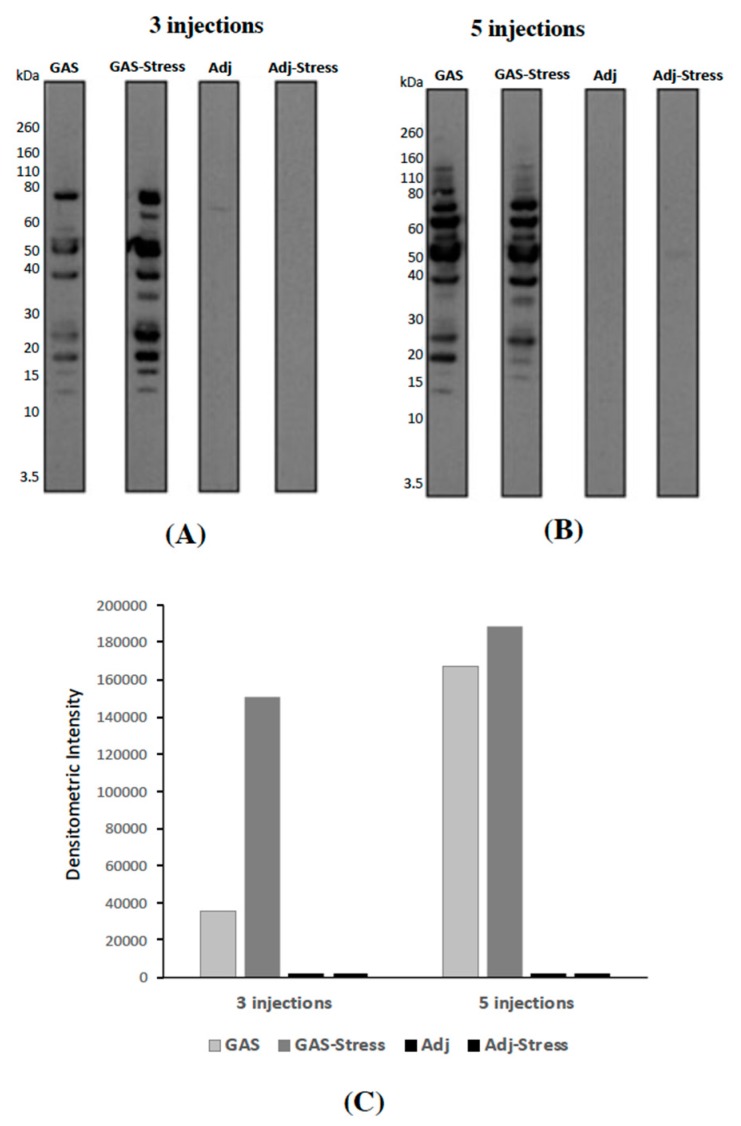
(**A**,**B**): Representative immunoblot of GAS extracts probed with pooled sera from mice either treated with 3 (**A**) or 5 doses (**B**) of GAS homogenate (GAS), GAS homogenate and psychosocial stress (GAS-Stress), adjuvant alone (Adj), or adjuvant and psychosocial stress (Adj-Stress). (**C**): Densitometric analysis of overall lane intensities from the blot shown in (**A**,**B**).

To investigate the presence of GAS-specific antibodies in sera from treated mice, we loaded 10 microliters of GAS homogenates onto SDS-PAGE, transferred to nitrocellulose, and tested with pools of sera from animals treated with three (Figure 2A) or five (Figure 2B) injections of: GAS homogenate (GAS), GAS homogenate and psychosocial stress (GAS-Stress), adjuvant alone (Adj), or adjuvant and psychosocial stress (Adj-Stress). Western Blot analyses of GAS homogenates using sera from animals treated with three (Figure 2A) or five (Figure 2B) injections of adjuvant (Adj and Adj-Stress mice) did not reveal any or very few bands. Several bands were instead detected in the lanes incubated with sera from animals receiving three (Figure 2A) or five (Figure 2B) GAS injections (GAS and GAS-Stress mice). These results indicate that sera from GAS treated mice recognize specific GAS proteins. Interestingly, this profile appeared much more marked as a consequence of the 5 injections (Figure 2C).

### 3.2. Consequences of Psychosocial Stress on Serum Corticosterone Concentrations and Glucocorticoid Receptor mRNA Levels in Hypothalamus and Hippocampus 

To evaluate the consequences of chronic psychosocial stress on HPA function, we evaluated serum basal corticosterone concentrations one week after the beginning of the psychosocial stress procedure. We observed that corticosterone concentrations were significantly reduced in all Stress-treated subjects irrespective of exposure to GAS (Stress condition: F(1,26) = 4.859, *p* < 0.036; no Stress group: 41.296 ± 4.236 ng/mL; Stress group: 28.754 ± 3.255 ng/mL; Cohen’s d: 3.320). 

Given the key role of glucocorticoid receptors (GRs) in regulating corticosterone secretion and mediating its effects on general metabolism, we analyzed the regulation of GRs in the hypothalamus and hippocampus of experimental subjects. These two limbic regions were selected due to their relevance in the response to peripheral immune activation and social stress, as well as in the behavioural abnormalities already evidenced in the PANDAS model [19]. 

**Figure 3:**
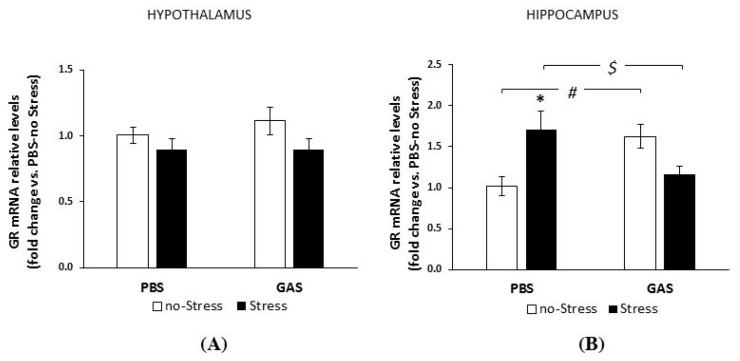
Glucocorticoid receptor (GR) mRNA expression in GAS-Stress mice and relative controls. RT-PCR were performed with mRNAs extracted from hypothalamus (**A**) and hippocampus (**B**) of control mice (PBS-no Stress), Group-A beta-haemolytic streptococcus injected mice (GAS-no Stress), psychosocial stressed mice (PBS-Stress), and mice exposed to haemolytic streptococcus and psychosocially stressed (GAS-Stress) at two weeks from treatment endings. Relative expression of GR mRNA in each area is presented as fold change over the expression measured in control mice (PBS-no Stress), taken as 1, and calculated using the 2-ΔΔCt method, normalized to hypoxanthine guanine phosphoribosyl transferase (HPRT), as detailed in the Materials and Methods section. Data are mean ± SEM, N= 4–6 per group. $ *p* < 0.05: GAS-Stress vs. PBS-Stress; # *p* < 0.05: GAS-no Stress vs. PBS-no Stress; * *p* < 0.05 PBS-no Stress vs. PBS-Stress. Cohen’s d measures are reported in Table S1A.

As shown in Figure 3A, hypothalamic mRNA GRs levels were indistinguishable across experimental groups two weeks after the end of treatments. 

Conversely, hippocampal GR mRNAs levels were affected by GAS treatment and chronic stress condition (GAS by Stress interaction, F(1,18) = 17.607, *p* = 0.001, Figure 3B). Specifically, both chronic stress and GAS exposure independently increased GR concentrations compared to PBS no-Stress subjects. Yet, experimental subjects exposed to both treatments at the same time were indistinguishable from PBS no-Stress controls. 

### 3.3. Behavioural Profile Exhibited during the First Active Social Confrontation

We have previously reported that the functional state of the HPA axis at the time of GAS exposure markedly affected GAS-induced neurobehavioral phenotype [19]. To deepen our investigation on the interaction between GAS exposure and environmental stress, we adopted a chronic psychosocial stress model [55]. Specifically, SJL male mice were randomly paired to resident males of the CD1 strain to attain exposure to a gradient of territorial aggression, reportedly high in CD1 [55,56,69]. Thus, half the mice exposed to repeated injections of either vehicle or GAS starting early in adolescence, were later on randomized to psychosocial stress during four weeks according to an established protocol [70]. The Stress condition started by maintaining the two pair members, which had agonistic confrontation on a daily basis, co-housed but prevented from physical interaction. According to the procedure, mice were indeed separated by a transparent and perforated partition allowing continuous sensory contact, thus mimicking a lifelong stress threat.

As reported in Table 1, data collected during the 1st active agonistic confrontation of the psychosocial stress procedure showed that GAS-Stress mice were exposed to significantly higher levels of aggressive behavior than PBS-Stress mice (Treatment by Time interaction (F(1,37) = 4.338, *p* = 0.044, for frequency; F(1,37) = 3.578, *p* = 0.066, for duration). Accordingly, GAS-Stress mice attained a Defensive upright posture more often and much earlier than controls (Treatment (F(1,37) = 3.545, *p* = 0.0676, for frequency; F(1,38) = 4.907, *p* = 0.0328, for latency). For Immobility attack-related, a Treatment (F(1,38) = 3.777, *p* = 0.0785, for frequency; F(1,36) = 3.279, *p* = 0.0785, duration, respectively), were evidenced with increased levels being characteristic of GAS-Stress subjects. A quite similar profile appeared for Immobility contact-related (Treatment: F(1,37) = 3.982, *p* = 0.0534 for frequency; F(1,37) = 5.402, *p* = 0.0257, for duration). 

T-maze test showed, in accordance with our predictions, that repeated GAS immunizations resulted in impaired spontaneous alternation (Treatment: F(1,35) = 28.326, *p* < 0.001). Specifically, regardless of exposure to stress, GAS treated mice exhibited reduced spontaneous alternations compared to controls. Furthermore, although exposure to stress apparently influenced the effects of GAS (Stress condition × Treatment: F(1,35) = 5.025, *p* = 0.0314), post-hoc tests failed to reveal significant differences between stressed and control individuals within the respective treatment group (PBS-no Stress: 78.020 ± 2.902; PBS- Stress: 60.937 ± 5.642; GAS-no Stress: 36.607 ± 7.359; GAS-Stress: 44.885 ± 4.377; values are means ± SD; N = 9–10 per group. Cohen’s *d* for PBS-no Stress, GAS-no Stress: 7.404; PBS-no Stress, GAS-Stress: 8.923; PBS-Stress, GAS-Stress: 3.179; GAS-no Stress, GAS-Stress: 1.367).

### 3.4. Chronic Psychosocial Stress Increased Inflammatory Genes Expression in the Brain of GAS Mice 

By using RT-PCR technique, we then investigated the relative levels of typical markers known to be regulated under stress and inflammatory conditions, and playing a central role in mechanisms of neuronal and synaptic plasticity, whose modulation could affect brain function and behaviour. Specifically, we addressed the regulation of the pro-inflammatory cytokines IL-1β and TNF-α, the immunomodulatory cytokine IL-10, the inflammatory/oxidative stress-related enzymes iNOS, Arg-1, MnSOD, and the macrophage/microglial marker CD11b, in the hypothalamus and hippocampus of the experimental subjects. As mentioned above, these two limbic regions were selected for their relevance in the response to peripheral immune activation and social stress, as well as in behavioural abnormalities already evidenced in the PANDAS model [19].

**Figure 4:**
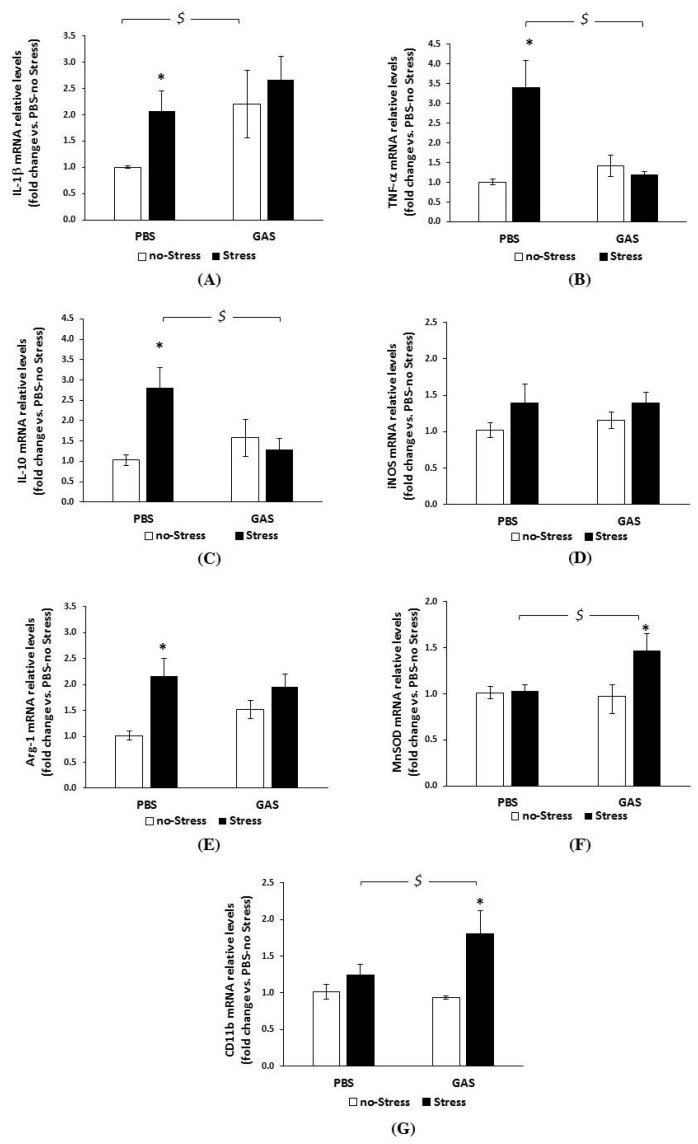
mRNA relative levels of inflammatory and oxidative stress-related markers in hypothalamus of GAS-Stress mice and relative controls. RT-PCR analysis was performed with mRNAs extracted from hypothalamus of control mice (PBS-no Stress), Group-A beta-haemolytic streptococcus injected mice (GAS-no Stress), psychosocially stressed mice (PBS-Stress) and mice exposed to haemolytic streptococcus and psychosocially stressed (GAS-Stress), at two weeks from treatment endings. Expression of each gene is presented as fold change over the expression measured in the hypothalamus of control mice (PBS-no Stress), taken as 1. The relative expression level of each mRNA was calculated using the 2-ΔΔCt method, normalized to hypoxanthine guanine phosphoribosyl transferase (HPRT), as detailed in the Materials and Methods section. Data are mean ± SEM, N = 4–6 per group. (**A**) IL-1β mRNA levels: $ *p* < 0.05 for GAS-no Stress vs. PBS-no Stress; * *p* < 0.05 for PBS-Stress vs. PBS-no Stress. (**B**) TNF-α mRNA levels: $ *p* < 0.05 for GAS-Stress vs. PBS-Stress; * *p* < 0.05 for PBS-Stress vs. PBS-no Stress. (**C**) IL-10 mRNA levels: $ *p* < 0.05 for GAS-Stress vs. PBS-Stress; * *p* < 0.05 for PBS-Stress vs. PBS-no Stress. (**D**) iNOS mRNA levels. (**E**) Arg-1 mRNA levels: * *p* < 0.01 for PBS-Stress vs. PBS-no Stress. (**F**) MnSOD mRNA level: $ *p* < 0.05 for GAS-Stress vs. PBS-Stress; * *p* < 0.05 for GAS-Stress vs. GAS-no Stress. (**G**) CD11b mRNA levels: $ *p* < 0.05 for GAS-Stress vs. PBS-Stress; * *p* < 0.05 for GAS-Stress vs. GAS-no Stress. Cohen’s d measures are reported in Table S1A.

**Figure 5:**
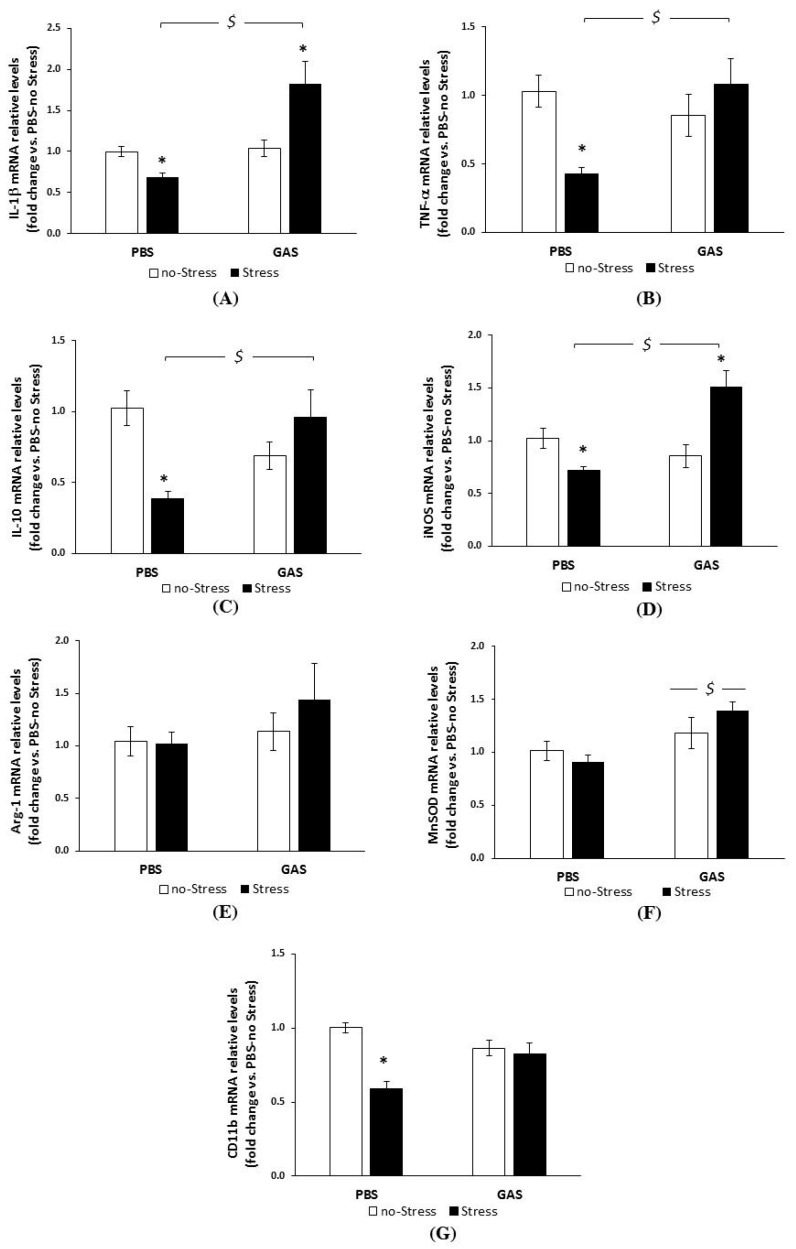
mRNA relative levels of inflammatory and oxidative stress-related markers in the hippocampus of GAS-Stress mice and relative controls. RT-PCR analysis was performed with mRNAs extracted from hippocampus of control mice (PBS-no Stress), Group-A beta-haemolytic streptococcus exposed mice (GAS-no Stress), psychosocially stressed mice (PBS-Stress) and mice exposed to haemolytic streptococcus and psychosocially stressed (GAS-Stress), at two weeks from treatment endings. Expression of each gene is presented as fold change over the expression measured in the hippocampus of control mice (PBS-no Stress), taken as 1. The relative expression level of each mRNA was calculated using the 2-ΔΔCt method, normalized to hypoxanthine guanine phosphoribosyl transferase (HPRT), as detailed in the Materials and Methods section. Data are mean ± SEM, *n* = 4–6 per group. (**A**) IL-1β mRNA levels: $ *p* < 0.01 for GAS- Stress vs. PBS-Stress; * *p* < 0.05 for GAS-Stress vs. GAS-no Stress and for PBS-Stress vs. PBS-no Stress. (**B**) TNF-α mRNA levels: $ *p* < 0.05 for GAS-Stress vs. PBS-Stress; * *p* < 0.05 for PBS-Stress vs. PBS-no Stress. (**C**) IL-10 mRNA levels: $ *p* < 0.05 for GAS-Stress vs. PBS-Stress; * *p* < 0.05 for PBS-Stress vs. PBS-no Stress. (**D**) iNOS mRNA level: $ *p* < 0.01 for GAS-Stress vs. PBS-Stress; * *p* < 0.01 for GAS-Stress vs. GAS-no Stress and *p* < 0.05 for PBS-Stress vs. PBS-no Stress. (**E**) Arg-1 mRNA levels. (**F**) MnSOD mRNA level: $ *p* < 0.05 for PBS vs. GAS. (**G**) CD11b mRNA levels: * *p* < 0.01 for PBS-Stress vs. PBS-no Stress and vs. GAS-Stress. Cohen’s d measures are reported in Table S1A.

All data (mean ± SEM) on transcript levels obtained two weeks after the end of treatment, alongside with the statistical analyses, are reported in Figure 4 (hypothalamus) and Figure 5 (hippocampus).

In the hypothalamus (Figure 4), ANOVA yielded a significant effect for GAS and Stress single treatments on IL-1β mRNA levels (panel A), with both treatments inducing increased IL-1β expression (GAS: F(1,15) = 5.863, *p* = 0.029; Stress: F(1,15) = 4.401, *p* = 0.053). The combination of GAS and Stress treatments did not add any change to the profile (F(1,15) = 0.723, *p* = 0.409).

As shown in Figure 4B, no changes in TNF-α mRNA levels due to GAS exposure (F(1,14) = 2.909, *p* = 0.110) were found. In contrast, PBS-Stress mice had higher transcript levels than PBS-no Stress mice (F(1,14) = 4.572, *p* = 0.05). Interestingly, the combination of GAS and Stress completely abated the up-regulatory effect of Stress alone (GAS by Stress interaction: F(1,14) = 7.597, *p* = 0.015).

A similar profile was found for IL-10 (Figure 4C): while GAS exposure per se did not modify IL-10 mRNA levels, Stress treatment upregulated the cytokine transcripts (F(1,14) = 2.558, *p* = 0.132) compared to no-Stress controls. Also in this case, the combination of GAS and Stress completely abated the upregulation induced by Stress alone (GAS by Stress interaction: F(1,14) = 6.248, *p* = 0.025). iNOS mRNA levels (Figure 4D) were neither modified by the two treatments *per se* nor by their interaction.

As shown in Figure 4E, no changes in Arg-1 mRNA levels due to GAS exposure were observed. In contrast, values for PBS-Stress mice were markedly increased (F(1,14) = 14.07, *p* = 0.002). In this case, we failed to observe any interaction between GAS and Stress (F(1,14) = 2.650, *p* = 0.125).

In the absence of a main effect of GAS on MnSOD mRNA levels (Figure 4F) ANOVA indicated that the Stress group as a whole was higher than control (F(1,13) = 5.692, *p* = 0.0329). Further, a significant GAS by Stress interaction (F(1,13) = 5.411, *p* = 0.0368) revealed that the upregulation of mRNA transcripts was specific to the GAS-Stress group. 

No main effect of GAS on CD11b (Figure 4G) mRNA levels was found. ANOVA yielded an effect of Stress as a whole (F(1,17) = 7.770, *p* = 0.0126), and a GAS by Stress interaction (F(1,17) = 2.727, *p* = 0.117); Tukey post hoc indicated that the upregulation of mRNA transcripts due to Stress was again specific to GAS-Stress mice. 

To summarize, two weeks after the last GAS injection, among the transcripts analysed in the hypothalamus, only IL-1β transcripts were reliably modified by GAS inoculation per se. Chronic psychosocial Stress per se upregulated both typical pro-inflammatory (IL-1β and TNF-α) and anti-inflammatory genes (IL-10 and Arg-1) while leaving unaltered the expression of the pro- and anti-oxidant enzymes iNOS and MnSOD and the phagocytic marker CD11b. When mice, previously inoculated with GAS, were faced with stress adverse experience, also MnSOD and CD11b resulted up-regulated. In contrast, TNF-α and IL-10 were down-regulated compared to Stress alone, suggesting the activation of the oxidative defense system in the combined condition, and a more pronounced or longer lasting inflammatory macrophage/microglial activation.

Noteworthy, in the hippocampus (Figure 5), GAS and Stress elicited a differential regulation of these inflammatory genes compared to the hypothalamus. Indeed, repeated GAS inoculation alone did not significantly or reliably alter the expression levels of the genes analysed (panels A–G). This suggests that, at this time point, the inflammatory reaction to the GAS stimulus was already subsided in this region, at least in term of the mRNA regulation of the panel of genes assayed.

Unlike GAS per se, Stress alone significantly reduced IL-1β, TNF-α, IL-10, iNOS, and CD11b mRNA levels (Figure 5, panels A–D, and G respectively), as revealed by post hoc analyses (see the Figure legend).

Interestingly, in the absence of significant GAS-related changes per se, the combination of GAS and Stress reverted the Stress-induced down-regulatory effect, indicating a relevant interaction of the two treatments. Indeed, GAS-Stress mice showed comparable TNF-α, IL-10, and CD11b levels than PBS-no Stress subjects, and higher IL-1β and iNOS levels (GAS by Stress interaction for TNF-α: F(1,19) = 9.695, *p* = 0.0057; for IL-10: F(1,16) = 14.502, *p* = 0.0015; for IL-1β: F(1,20) = 16.754, *p* = 0.0006; for iNOS: F(1,20) = 24.628, *p* < 0.0001; for CD11b: F(1,19) = 16.789, *p* = 0.0006). 

Unlike the other genes analyzed, Arg-1 mRNA levels (Figure 5E) were unaffected, irrespective of both treatments and their combination. 

Considering MnSOD expression (Figure 5F), ANOVA yielded a significant effect for GAS exposure (F(1,15) = 9.999, *p* = 0.006), with GAS group as a whole being higher than controls (for post hoc, see figure legend). Stress *per se* or its combination with previous GAS inoculation, did not affect the profile. 

As a whole, these data suggest that Psychosocial Stress mostly induced a down-regulated basal immune profile at the hippocampal level, while the combination with GAS exposure was associated with an increased, longer-lasting, pro-inflammatory oxidative condition in the hippocampus.

### 3.5. Psychosocial Stress Affects Mitochondrial OXPHOS Machinery and Reduces Energy Status in the Brain of GAS Immunized Mice

**Figure 6:**
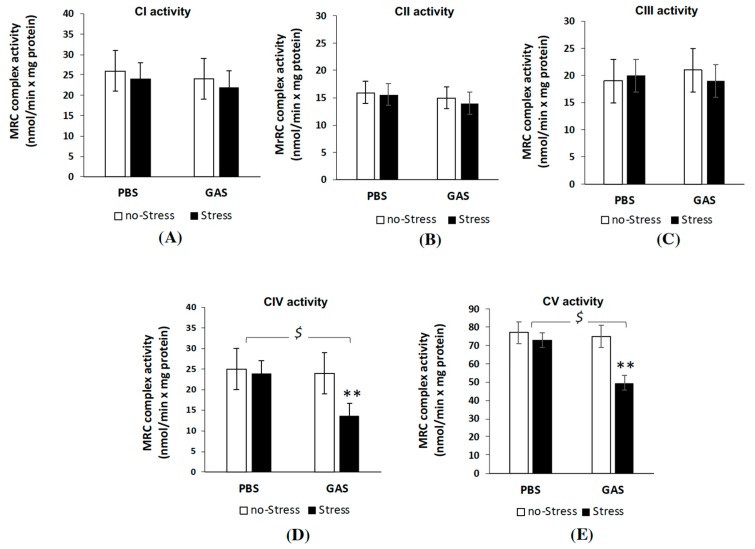
Mitochondrial respiratory chain (MRC) complex activities in brain of GAS-Stress mice and relative controls. The activities of the MRC (**A**) complex I, (**B**) complex II, (**C**) complex III, (**D**) complex IV and (**E**) complex V (ATP synthase) were measured spectrophotometrically in mitochondrial membrane enriched fractions from cryopreserved brain hemispheres of wt littermates control mice (PBS-no Stress), Group-A beta-haemolytic streptococcus exposed mice (GAS-no Stress), psychosocially stressed mice (PBS-Stress) and psychosocially stressed mice exposed to haemolytic streptococcus (GAS-Stress), at two weeks from treatment endings. Complex activities are expressed as nmol/min × mg protein. Data are mean rates ± SD obtained from three independent experiments. For MRC complex IV and V activities $ *p* < 0.05 for PBS-Stress vs. GAS-Stress; ** *p* < 0.01 for GAS no-Stress vs. GAS-Stress. Cohen’s d measures are reported in Table S1B.

We first examined whether GAS and Stress treatments, separately or in interaction, could affect the MRC complex activity in mice brain mitochondria (Figure 6). Measurements of MRC complex I-IV as well as ATP synthase (complex V) activities showed no significant changes in all MRC activities in both PBS-Stress and GAS-no Stress treated mice, respect to control (PBS-no Stress) group. Interestingly, GAS treatment in mice exposed to psychosocial stress (GAS-Stress group) resulted in a significant reduction in the activity of complex IV and V compared to control mice (F(1,12) = 16.12, *p* = 0.002; F(1,12) = 95.28, *p* = 0.001, respectively). Importantly, chronic stress plus GAS treatments did not alter the mitochondrial content in the brain tissue being 4.70 ± 0.6 and 4.66 ± 0.3 mg the amount of mitochondrial proteins obtained respectively from untreated and GAS-Stress groups (*p* > 0.5), from brain hemisphere tissues with comparable wet weights (0.24 ± 0.1 g).

**Figure 7:**
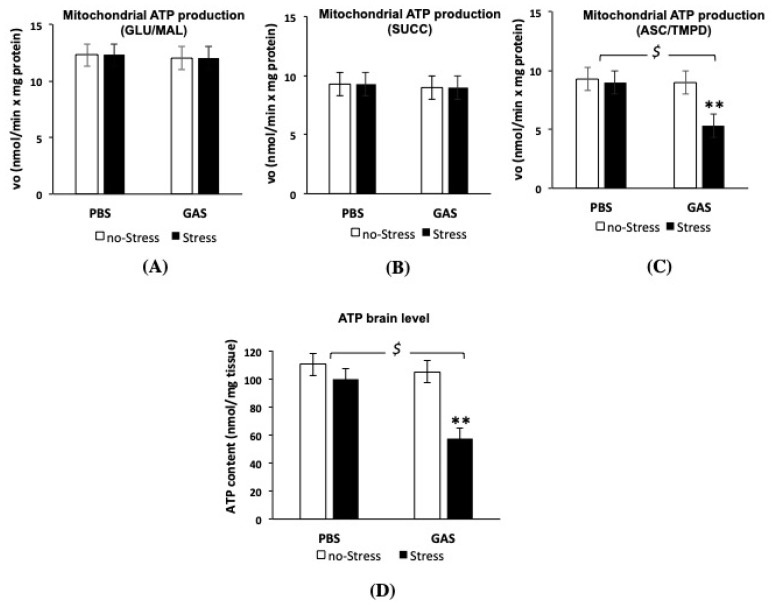
Mitochondrial ATP production and ATP level in the brain of GAS-Stress mice and relative controls. The rate of mitochondrial ATP production was measured in mitochondria isolated from cryopreserved brain hemispheres in the presence of the respiratory substrates of (**A**) complex I glutamate plus malate (GLU/MAL), (**B**) complex II succinate (SUCC) plus rotenone or (**C**) complex IV ascorbate plus TMPD (ASC/TMPD). Values are mean rates ± SD obtained from three independent experiments and expressed as nmol/min × mg protein. Values are mean rates ± SD obtained from three independent experiments and expressed as nmol/min × mg protein. (**D**) The ATP level was measured as described in Material and Methods. Values are mean rates ± SD obtained from three independent experiments and expressed as nmol/mg protein. For the panels C and D $ *p* < 0.05 for PBS-Stress vs. GAS-Stress. ** *p* < 0.01 for GAS no-Stress vs. GAS-Stress. Cohen’s d measures are reported in Table S1C.

In order to investigate whether the MRC defective complex activities found in GAS-Stress mice group was accompanied by an impairment of bioenergetic efficiency, the mitochondrial ATP synthesis was measured following the relative contribution of the individual MRC complexes of the OXPHOS apparatus in the mitochondrial ATP production i.e. by adding the respiratory substrates of either complex I (GLU/MAL), complex II (SUCC) or complex IV (ASC/TMPD), as energy sources (Figure 7). Consistently with the data obtained from MRC activity measurements, GAS-Stress mice showed a significant reduction in mitochondrial ATP synthesis only when using as energy source the respiratory substrates of complex IV (Figure 7C); after post hoc comparison on GAS-Stress vs. PBS-no Stress mice: F(1,12) = 4.60, *p* = 0.04). No significant differences among all mice groups were found in the complex I- and II-dependent rate of mitochondrial ATP production from brain mitochondria. These results suggest that specific components of the respiratory apparatus resulted selectively affected by GAS in psychosocial stressed mice and contributed to the shortage in mitochondrial ATP production. 

Interestingly, the levels of ATP assayed in the brain of all four groups were strongly lowered in GAS-Stress group as compared to the other groups (Figure 7D, F(1,16) = 18.36, *p* = 0.001), thus suggesting that alterations in mitochondrial ATP production by GAS in psychosocial stress conditions affected the whole brain energy status.

## 4. Discussion

The validated translational mouse model of PANDAS, adopted in the present study, has been extensively characterized from a behavioural and biochemical point of view in our previous studies [18,19]. Consistently with the hypothesis of the infectious and autoimmune pathogenesis of PANDAS (see [71]), we reported that repeated exposures to GAS induce an antibody-mediated response (also confirmed in the present study) and behavioural alterations homologous to clinical symptoms observed in PANDAS (impaired sensorimotor gating, and abnormal repetitive and perseverative behaviours). The behavioural phenotypes exhibited by GAS mice represent the preclinical analogue of core clinical symptoms observed in PANDAS and obsessive-compulsive syndrome [11,72,73], and are useful for studying their neurobiological basis. For example, increased behavioural rigidity, reflected in impairments in spontaneous alternations, could be due to alterations in forebrain structures (prefrontal cortex and dorsal striatum) [74] and imbalances in dopaminergic [75] and serotonergic [76] neurochemical systems.

We previously reported that the behavioural changes observed in GAS mice were associated with immune-mediated brain alterations, as indicated by the presence of inflammatory infiltrates and activated microglia at the level of the rostral diencephalon (see [19,71] for a detailed discussion). In the same rodent model, we observed that neonatal corticosterone administration contrasted both behavioural and immunohistochemical alterations induced by later GAS exposure. These compensatory effects co-occurred with persistent modifications in HPA activity and remarkable plasma increases of several cytokines and chemokines, supporting the view that the HPA axis may contribute to the regulation of the immune responses involved in the pathological sequelae of PANDAS and ultimately modulate the severity of the PANDAS-related phenotype. 

On this basis, herein, we further characterized the sequelae of repeated exposures to a GAS homogenate during development (between late infancy and young adulthood) on behaviour, neuroinflammatory and brain oxidative stress responses, and mitochondrial functional aspects later at adulthood, and addressed the modulatory effects of chronic psychosocial stress on the same parameters. 

With the aim of a translational approach, and to model a chronic psychosocial stress condition [55,56,69], GAS mice and their controls were exposed to a gradient of territorial aggression by resident male mice. Specifically, compared to PBS-injected control mice, during the first active social confrontation, GAS mice were the recipients of consistently higher levels of aggressive behavior (in terms of Attacks received). GAS mice also showed a characteristic behavioural repertoire, consisting of a shortened latency to and an increased frequency of defensive upright postures, and time spent in immobility. The observed behavioral profile of chronic stress condition is consistent with recent literature on this translational model of adverse emotional experience [70], and on the reported interaction of chronic stress with central immune dysregulation [77]. We believe that this profile of increased aggression received by GAS mice during the first confrontation may relate to the fact that the homogenate injection resulted in an overt inflammatory profile. The latter may have signalled a state of vulnerability to the resident mouse which, in turn, may have increased its degree of aggression. Whilst this aspect is worth additional investigation, we note that the differential attacks received by Stress and GAS mice during the first day have unlikely extended to the following days of stress exposure. This tenet stems from the fact that, after the first day, during the following days, direct attacks were physically prevented by the experimenter, which interrupted the session upon the first occurrence of aggressive interaction. Ultimately, although future studies are needed to clarify this aspect, we suggest that the increased aggression received by GAS mice on day one may be due to a short-term effect of GAS homogenate on individual phenotype, but that – in the light of the experimental paradigm adopted—such differential profile is unlikely to explain the observed findings.

Consistently with our previous histochemical observations, suggestive of GAS-induced central immune activation, here we found increased hypothalamic mRNA levels of the pro-inflammatory cytokine IL-1β in GAS mice, analysed two weeks after the last GAS inoculation, revealing a long-lasting central inflammatory effect of peripheral immunization. However, the other inflammatory- or oxidative stress-related genes analysed (i.e. TNF-α, IL-10, iNOS, Arg-1, MnSOD, CD11b) were not altered compared to PBS-injected control subjects. 

Interestingly, at the hippocampal level, the expression of IL-1β was not reliably modified by GAS treatment per se, as well as that of TNF-α, iNOS, and MnSOD, while the immunomodulatory cytokine IL-10 and the macrophage/microglial phagocytic marker CD11b showed a tendency to decrease compared to PBS-injected control mice. 

It is worth noting that, as the above mRNA expression analyses were conducted two weeks after the last GAS inoculation, our data depict the long-lasting alteration of neuroinflammatory state consequent to repeated GAS challenges rather than the acute alteration of the genes analysed. It is well known that brain immune cells (mainly microglia and astrocytes) undergo a profound rearrangement of their functions following chronic stimulation, and acute and chronic preconditioning regimens differentially affect their responsiveness to a later inflammatory challenge, for the onset of distinct mechanisms of molecular memory ([78,79] and refs therein). Therefore, the mRNA data of inflammatory markers reflect brain immune cell adaptation to a chronic stimulation. Similar considerations apply to the analyses of inflammation-related transcripts in subjects exposed to Psychosocial Stress, two weeks after treatment termination.

Also in this case, and in line with previous experimental studies on Psychosocial Stress effects [77], we observed a region-specific modulation of central cytokine expression. The direction of mRNA regulation by Psychosocial Stress was however different from what found for GAS exposure, as it was characterized by a prominent inflammatory response in the hypothalamus, and an opposite profile in the hippocampus. Specifically, compared to PBS-injected control mice, while Stress upregulated the mRNA levels of the inflammatory genes IL-1β, TNF-α, IL-10, Arg-1, and marginally modulated iNOS and CD11b mRNAs in the hypothalamus, it downregulated the same genes (with the exception of Arg-1) in the hippocampus. 

Besides their role in neuroinflammatory processes, cytokines typically participate in brain development and plasticity, by translating environmental inputs into molecular signals [80]. An imbalance between pro-inflammatory and anti-inflammatory cytokines can lead to long-lasting changes in brain anatomy and function, and therefore long-term impairments in mood, cognition, and behavior [81]. We did not analyze possible changes in brain anatomy in the GAS mouse model, but the neuroimmune and behavioural alterations found in our study further support the link between inflammatory gene regulation and behaviour. 

Obvious limitations of mRNA analysis on bulk hippocampus and hypothalamus include the possible dilution of signals confined to specific sub-regions important for immune and stress responses, as well as the exclusion of additional levels of gene expression regulation; nonetheless, our data clearly indicate that GAS peripheral infection and Stress have long-term and region-specific consequences on brain immune homeostasis.

Region-specific patterns of up-regulation of distinct cytokines and differences in the extent and time-course of activation in response to peripheral and central stressors have been reported in different experimental models, albeit mechanisms conferring specificity of action remain to be fully elucidated. Among the different factors accounting for these differences, neutrophil infiltration rate, microglia/astrocyte density, blood brain barrier permeability, and relative densities of mineralocorticoid and glucocorticoid receptors may represent valid targets [82,83,84]. 

Although the identification of possible mechanisms is far beyond the scope of the present experimental investigation, the finding that GAS and chronic psychosocial Stress independently upregulated GR expression at the hippocampal but not hypothalamic level suggests that these changes might be involved in the different sensitivity of the two brain regions to HPA-related regulatory mechanisms of inflammation. This is consistent with other reports from different chronic stress models [85,86]. 

Furthermore, while both repeated GAS exposure and Psychosocial Stress exerted independent effects, the main translational finding of the present study resides in the fact that the latter exacerbated the effects of the former. In accordance with experimental data indicating that variations in circulating corticosteroids may influence autoimmune phenomena [87,88], we observed that chronic psychosocial stress, which exerted persistent effects on HPA axis activity (revealed by changes in hippocampal GR expression and reduced peripheral corticosterone concentrations), exacerbated the behavioural, immune and mitochondrial effects of GAS administration. The observation that the combination of GAS and Stress halted the upregulation of GR in the hippocampus, together with the reduced corticosterone concentrations found in this experimental group, suggest a persistent blunted HPA activity in these mice at the time point of our analyses. 

Classical studies conducted by Levine and his group showed that psychological and physiological stress suppresses [87] and adrenalectomy potentiates [89] vulnerability to experimental autoimmune encephalomyelitis. Ultimately, it is tenable that, depending on the directionality of the long-term consequences exerted by experimental manipulations on HPA activity (increase or decrease in circulating concentrations of corticosteroids and regulation of their receptors), autoimmune responses may be either potentiated or contrasted. Complex feedforward and feedback control mechanisms of gene expression between glucocorticoids, activating GRs, and cytokines (such as IL-1β and TNF-α) have been described in different experimental models [90,91] providing an explanation for the not univocal role of glucocorticoids on inflammatory gene expression regulation. Further investigations will be needed to address these issues in our GAS-Stress model and to dissect the interactions that can take place in the two separate or combined GAS and Stress conditions.

Interestingly, the combination of GAS exposures with chronic stress-induced a significant upregulation of the hippocampal levels of IL-1β, TNF-α, and the inducible enzyme iNOS compared to PBS-injected control subjects, which was not achieved by the single treatments. GAS-Stress treatment also elicited a further upregulation of CD11b mRNA compared to Stress (in the hippocampus and hypothalamus) or GAS alone (in the hypothalamus), suggesting an increased macrophage/microglia activation in these areas. 

Overall, these findings are in line with growing evidence indicating that cross-sensitization can occur between immune-induced and stress-induced pro-inflammatory cytokines, resulting in the potentiation of CNS cytokine responses [37,38,39,40]. The phenomenon of cross-sensitization suggests that a shared neural substrate, mainly identified by others in the primary immune effector cell in the nervous system i.e. microglia, may be primed by either stress or immune activation [37,38,40,92]. Whatever the underlying mechanisms may be, cross-sensitization provides a mechanism explaining how stress can exacerbate inflammatory disease processes and vice versa. 

In consideration of the increasingly recognized modulatory action of cytokines and nitric oxide on mechanisms of neuronal and synaptic plasticity, our data support the view that peripheral inflammation and stress converge on pathways culminating in disruption of brain homeostatic functions and neuroinflammation. In addition, by the generation of nitrogen reactive species, iNOS can contribute to oxidative stress as shown in different tissues and experimental models [93,94], including psychogenic stress treatments [95,96].

Consistently, we observed that GAS-Stress treatment promoted the expression of MnSOD—the primary antioxidant enzyme in mitochondria—at hippocampal and hypothalamic levels; these data indicate the induction of the antioxidant defense system. MnSOD, a key component of the enzymatic antioxidant system, is upregulated by various mediators of oxidative stress, including reactive oxygen and nitrogen species and inflammatory cytokines, such as IL-1β and TNF-α [49], and its abnormalities have been documented in several clinical cases and experimental neurodegenerative processes [97]. 

Superoxide dismutase function is activated in the mitochondria to detoxify free radical superoxide anion with formation of less reactive peroxide anion (H_2_O_2_) [49]. However, conditions of chronic increase of MnSOD activity could result in H_2_O_2_ accumulation, thereby causing mitochondrial alterations. In particular, the MRC complex IV-cytochrome c oxidase is a target of hydrogen peroxide showing various sites of oxidative modifications, which leads to a decline in its catalytic activity [98].

Consistently with a condition of oxidative stress in the GAS-Stress mouse model, our data demonstrate that the combined exposure to GAS and stress caused a reduction of the complex IV activity and decreased complex IV-dependent ATP production. These deficits, together with a reduced ATP synthase activity, impair mitochondrial bioenergetics resulting in a deficit of brain ATP content. The defective whole brain energy status and activation of MnSOD were not elicited by the single treatments, suggesting for the first time a direct link between inflammation, mitochondrial bioenergetic deficiency and ROS production in the combined infection/stress condition. 

This is, to the best of our knowledge, the first report suggesting a clear link between inflammation status and mitochondrial dysfunction in infectious GAS condition exacerbated by psychosocial stress. 

Mitochondrial dysfunction is emerging as a pathological mechanism underlying various inflammatory and autoimmune diseases, which become worse when accompanied by systemic inflammation and oxidative stress [99]. Accumulating clinical and preclinical evidence indicate that mitochondria are key players in neuroinflammatory and neurodegenerative diseases, as well as a critical intersection point connecting early-life stress, brain programming and mental health [100,101,102]. There is growing evidence for the involvement of both mitochondrial ROS and mitochondrial metabolism in inflammatory microglia/macrophage activation [47,103,104]. Moreover, it has been recently shown that alterations of mitochondrial activity in microglia hamper the process of alternative activation, suggesting that in severe clinical neurological conditions characterized by mitochondrial dysfunctions, microglia may not be able to induce a full anti-inflammatory response, exacerbating neuroinflammation [48]. 

Our results in the preclinical model of PANDAS substantiate the translational hypothesis that Streptococcus infection could induce an inflammatory status in PANDS patients, exacerbated by stress conditions, with the secretion of inflammatory cytokines, which likely induce increase of ROS production and mitochondrial dysfunction, resulting in brain energy deficit, which in turn intensify the clinical symptoms severity. Previous clinical studies have demonstrated that inflammatory cytokines such as interleukin-17 disable the main function of mitochondria, the energy production by respiration, and activate autophagy in an autoimmune disease such as rheumatoid arthritis [51]. Indeed, the critical role of pro-inflammatory cytokines on mitochondrial stress signalling and proteostasis is well known [105,106]. Of note, a direct link between stress-derived corticosteroids and mitochondrial function has been demonstrated in recent studies, revealing that activated GRs, besides their genomic action, can translocate to the mitochondrial compartment and regulate mitochondrial mRNA expression, including complex 1 subunits and ATP-synthase 6 expression [107,108]. The possible regulatory function of GRs on mitochondrial activities in the GAS-Stress model will deserve further investigations.

## 5. Conclusions

Our results demonstrate that chronic psychosocial stress, which per se altered the expression of neuroinflammatory markers in the hippocampal and hypothalamic regions, exacerbated the neuroinflammatory alterations induced by experimental GAS exposures in the same areas. In addition, the combined GAS/Stress treatment elicited mitochondrial dysfunctions, brain energy deficit and upregulation of manganese superoxide dismutase (MnSOD), a mitochondrial enzyme playing a major role in modulation of mitochondrial oxidative stress.

Our findings demonstrate the negative impact of social stress on PANDAS symptomatology and provide a functional explanation to epidemiological and clinical data as well as a biological platform to investigate the impact of psychosocial stress on immune-related clinical neurological diseases.

In this study, we offered a proof of principle and a translational hypothesis that experimentally-induced alterations of HPA functionality may calibrate the individual response and vulnerability to autoimmune phenomena. Furthermore, we identified a potential translational link between environmental stress experience and the underlying mechanisms (promotion of immunomodulatory processes) capable of promoting/exacerbating the progression of the pathological phenotype. We propose that these data may inform future clinical strategies in the treatment of PANDAS. 

## Figures and Tables

**Table 1:** Analysis of the behavioural profile during the 1st active agonist confrontation in the psychosocial stress procedure. Submissive behaviours = crouched posture + submissive posture. Duration = time spent (s) performing the behaviour, expressed as a mean of two 5-min intervals. Values are means ± SD; N = 9–10 per group. * *p* < 0.05, ^ trend, df = degrees of freedom.

Behaviour	Parameter	GAS-Stress	PBS-Stress	F (df)	*p*	*Cohen’s d*
**Attack received**	Duration	6.559 ± 1.135	4.996 ± 0.957	3.578 (1,37)	0.07 Treat × Time ^	1.489
	Frequency	9.974 ± 1.866	7.175 ± 1.422	4.338 (1,37)	0.04 Treat × Time *	1.687
**Defensive upright posture**	Duration	23.724 ± 3.616	16.925 ± 3.482	1.707 (1,38)	0.20	1.915
	Frequency	10.575 ± 1.729	6.079 ± 1.076	3.545 (1,37)	0.07 Treat ^	3.122
	Latency	212.518 ± 36.059	337.022 ± 43.116	4.907 (1,38)	0.03 Treat *	3.132
**Self-grooming**	Duration	13.048 ± 2.366	12.784 ± 2.318	0.007 (1,38)	0.93	0.113
	Frequency	3.350 ± 0.452	2.900 ± 0.477	0.707 (1,38)	0.41	0.968
**Immobility attack-related**	Duration	8.967 ± 2.156	2.923 ±.1.181	3.279 (1,36)	0.08 Treat ^	3.477
	Frequency	3.475 ± 0.791	2.350 ± 0.648	3.777 (1,38)	0.06 Treat ^	1.556
**Immobility contact-related**	Duration	13.3 ± 3.650	3.988 ± 1.022	5.402 (1,37)	0.03 Treat *	3.474
	Frequency	2.950 ± 0.636	1.447 ± 0.375	3.982 (1,37)	0.05 Treat ^	2.879
**Submissive behaviours **	Duration	55.198 ± 6.601	45.790 ± 7.840	0.843 (1,38)	0.36	1.298
	Frequency	24.550 ± 3.890	17.700 ± 3.069	1.911 (1,38)	0.17	1.955
**Fleeing**	Duration	7.909 ± 1.452	6.480 ± 1.233	0.381 (1,37)	0.54	1.061
	Frequency	9.650 ± 1.743	6.350 ± 1.212	1.607 (1,38)	0.21	2.198
	Latency	285.654 ± 39.534	349.358 ± 46.590	1.087 (1,38)	0.30	1.474

## Supplementary Materials

The following are available online at https://www.mdpi.com/2077-0383/8/10/1514/s1, Table S1: Effect Size measures (Cohen’s d) for the comparisons shown in Figure 3, Figure 4, Figure 5 (**A**), Figure 6 (**B**) and Figure 7 (**C**). * Cohen’s *d* corresponding to significant comparisons.
