# Supplementary material for: Brain-Immune Alterations and Mitochondrial Dysfunctions in a Mouse Model of Paediatric Autoimmune Disorder Associated with Streptococcus: Exacerbation by Chronic Psychosocial Stress
Source: J Clin Med. 2019 Sep 20;8(10):1514. doi: 10.3390/jcm8101514 (PMC6833026; doi:10.3390/jcm8101514)
Supplement: Supplementary file 1 [file jcm-08-01514-s001.pdf]

Table S1
Effect Size measures (Cohen's *d*) for the comparisons shown in Figures 3–5 (**A**), 6 (**B**), and 7 (**C**).
***** Cohen's *d* corresponding to significant comparisons.

A

**Cohen's *d* for results shown in Figures 3, 4 and 5:**								
**mRNA**	**PBS-Stress *vs* PBS-no Stress**		**GAS-no Stress *vs* PBS-no Stress**		**GAS-Stress *vs* PBS-Stress**		**GAS-Stress *vs* GAS-no Stress**	
	**Hypothal**	**Hippoc**	**Hypothal**	**Hippoc**	**Hypothal**	**Hippoc**	**Hypothal**	**Hippoc**
**GR**	0.454	1.870 *	1.765	2.153 *	0.000	1.481 *	1.897	1.673 *
**IL-1β**	1.798 *	2.395 *	1.486 *	0.167	0.711	2.671 *	0.470	1.744 *
**TNF-α**	1.822 *	2.838 *	1.169	0.484	1.668*	2.254 *	0.714	0.585
**IL-10**	1.746 *	3.391 *	0.973	1.613	1.410*	1.810 *	0.517	0.822
**iNOS**	0.779	1.747 *	0.693	0.639	0.006	3.264 *	0.833	2.309 *
**Arg-1**	2.257 *	0.058	2.010	0.229	0.328	0.683	1.268	0.473
**MnSOD**	0.057	0.861	1.310	0.561	1.274 *	3.083 *	1.731 *	0.799
**CD11b**	0.786	4.476 *	0.526	1.362	1.018 *	1.984 *	1.714 *	0.348

B

	**Cohen's *d* for results in Figure 6**	
**MRC complex**	**GAS-Stress *vs* PBS-Stress**	**GAS-Stress *vs* GAS-no Stress**
**Complex I**	0.283	0.500
**Complex II**	0.256	0.548
**Complex III**	0.580	0.283
**Complex IV**	7.983 *	4.360 *
**Complex V**	12.670 *	10.063 *

C

	**Cohen's *d* for results in Figure 7**	
**ATP production**	**GAS-Stress *vs* PBS-Stress**	**GAS-Stress *vs* GAS-no Stress**
**GLU/MAL**	0.225	0.169
**SUCC**	0.063	0.013
**ASC/TMPD**	2.076 *	2.879 *
**ATP brain level**	3.563 *	2.870 *
